# Expired Patents

**Published:** 1906-05

**Authors:** 


### EXPIRED PATENTS.

February 19, 1906.

397990, Dental engine, I. G. Leek, San Francisco, Cal.

March 5, 1906.

399071, Combination dental tool, J. J. R. Patrick, Belleville, Illinois.

399177, Dental tooth regulating screw, Ed. H. Angle, Minneapolis, Minn.

March 12, 1906.

399350, Dental dish holder, H. H. Sisson, New York, N. Y.

399406, Dentists' lathe, N. W. Holt, Bennington, Vt.

April 2, 1906.

400585, Method of protecting dental fillings, D. C. McNaughton, Jersey City, N. J.

400586, Dental syringe, D. C. McNaughton, Jersey City, N. J.

400587, Dental matrix, C. A. Meister, Allentown, Pa.

400713, Dental articulator, W. F. Slack and G. A. Smith, Lawrence, Mass.

400771, Dental rubber dam clamp, J. W. Ivory, Philadelphia, Pennsylvania.

April 9, 1906.

400921, Artificial tooth, Chas. H. Land, Detroit, Mich.

April 16, 1906.

401391, Regulating valve for dental vulcanizers, L. Struck, Hart, Mich.

